# Supplementary material for: A cytosolic reductase pathway is required for efficient N-glycosylation of an STT3B-dependent acceptor site
Source: J Cell Sci. 2021 Nov 25;134(22):jcs259340. doi: 10.1242/jcs.259340 (PMC8645230; doi:10.1242/jcs.259340)
Supplement: Reviewer comments [file joces-134-259340_review_history.pdf]

A cytosolic reductase pathway is required for efficient N-glycosylation of an STT3B-dependent acceptor site

Marcel van Lith, Marie Anne Pringle, Bethany Fleming, Giorgia Gaeta, Jisu Im, Reid Gilmore and Neil J. Bulleid
DOI: 10.1242/jcs.259340

Editor: Jennifer Lippincott-Schwartz

Review timeline

Original submission:	31 August 2021
Editorial decision:	7 October 2021
First revision received:	19 October 2021
Accepted:	27 October 2021

Original submission

First decision letter

MS ID#: JOCES/2021/259340

MS TITLE: A Cytosolic Reductase Pathway is Required for Complete N-Glycosylation of an STT3B-Dependent Acceptor Site.

AUTHORS: Marcel van Lith, Marie Anne Pringle, Bethany Fleming, Giorgia Gaeta, Jisu Im, James Reid Gilmore, and Neil J Bulleid
ARTICLE TYPE: Research Article

We have now reached a decision on the above manuscript.

To see the reviewers' reports and a copy of this decision letter, please go to: https://submit-jcs.biologists.org and click on the 'Manuscripts with Decisions' queue in the Author Area. (Corresponding author only has access to reviews.)

As you will see, the reviewers raise a number of substantial criticisms that prevent me from accepting the paper at this stage. They suggest, however, that a revised version might prove acceptable, if you can address their concerns. If you think that you can deal satisfactorily with the criticisms on revision, I would be pleased to see a revised manuscript. We would then return it to the reviewers.

We are aware that you may be experiencing disruption to the normal running of your lab that makes experimental revisions challenging. If it would be helpful, we encourage you to contact us to discuss your revision in greater detail. Please send us a point-by-point response indicating where you are able to address concerns raised (either experimentally or by changes to the text) and where you will not be able to do so within the normal timeframe of a revision. We will then provide further guidance. Please also note that we are happy to extend revision timeframes as necessary.

Please ensure that you clearly highlight all changes made in the revised manuscript. Please avoid using 'Tracked changes' in Word files as these are lost in PDF conversion.

© 2021. Published by The Company of Biologists under the terms of the Creative Commons Attribution License (https://creativecommons.org/licenses/by/4.0/).

I should be grateful if you would also provide a point-by-point response detailing how you have dealt with the points raised by the reviewers in the 'Response to Reviewers' box. Please attend to all of the reviewers' comments. If you do not agree with any of their criticisms or suggestions please explain clearly why this is so.

Reviewer 1

Advance summary and potential significance to field

The work described by van Lith et al identifies the contribution of a cytosolic reductase pathway to the efficient N-glycosylation of a specific acceptor site in the model substrate hemopexin. The experimental data are robust and well controlled and the authors provide a convincingly level of molecular insight into the process that they identify. Their work underlines the complexity of N-glycosylation and the way in which the effectiveness of this ER luminal process can be influenced by events occurring in the cytosol.

Comments for the author

I have raised a few minor points, as detailed below, that I believe the authors can successfully address by re-writing the relevant text. Alternatively they might wish to provide additional experimental data, but I do not consider this to be in any way essential.

Minor points:
1. The title refers to “complete N-glycosylation” of an acceptor site, the quantification data included in the manuscript show this is rarely if ever achieved in their in vitro system. I suggest the title is changed to “required for efficient N-glycosylation” or some similar term.

2. Following on from point 1, can the authors clarify whether existing literature indicates that a fraction of hemopexin is hypoglycosylated in vivo under “normal” conditions, i.e. is it a tricky substrate anyway?

3. Line 120. The authors should point out that it is a minor fraction of hemopexin that is hypoglycosylated under the conditions described. This fraction varies, but in each case the majority of the translocated protein is fully N-glycosylated.

4. At various points where the authors state that reagents like G6-P reverse the hypoglycosylation of N-187 this is true in the sense that the level of under-glycosylated material is reduced but it is never completely absent. The term partially reversed would seem to be more appropriate here and indeed the authors use the phrase “partially resolve the hypoglycosylation” in line 286 of their manuscript in a similar context.

5. The authors use protease protection to show the circa 50 kDa products is non-translocated precursor protein. Do the authors have any suggestions as to why G6P would reduce the amount of this species.
Likewise, in the case of the the single cysteine mutant, C188-RC, there is a substantial increase in the amount of this non-translocated species? Is the mutant partially defective in membrane translocation?

6. Figure 4B. There seems to be a general loss of signal when samples are treated with PK in the presence of G6-P? Any thoughts?

7. The data shown in Figure 6 are clear cut and direct us to STT3B complex and its MagT1/TUSC3 subunits. In panel 6B I can see an additional faint band appearing (as compared to panel 6A) which runs between the hypoglycosylated hemopexin and its non-translocated precursor as defined above. Does this mean that an additional sequon is now being partially under-glycosylated when the STT3B complex is defective?

© 2021. Published by The Company of Biologists under the terms of the Creative Commons Attribution License (https://creativecommons.org/licenses/by/4.0/).

8. I would encourage the authors to include a simple schematic or cartoon that outlines the two proposed potential fates of N-187 when a disulphide is formed before or after its arrival at the STT3A complex. I think this would be very helpful for many readers.

Reviewer 2

Advance summary and potential significance to field

In this study, van Lith et al. have investigated the link between cytosolic redox conditions, ER redox conditions, disulfide formation, and efficiency of glycosylation in the ER. First, they focus on a model substrate to study this link, Hemopexin, and then further investigate an observed hypoglycosylation of N187 that is resolved upon altering cytosolic redox conditions—through the addition of exogenous G6-P to their in-vitro translation system. This hypoglycosytation of N187 is proven to be dependent on the presence of C188 leading the authors to investigate a potential role for disulfide formation and accessibility of the glycosylation sequon. Finally, the authors probe how the cytosolic-ER redox conditions might be coupled through KO of glycosylation machinery, or multiple thiol reductases.

The major conceptual advance at play seems to be determining the mechanism by which cytosolic redox state and ER redox state are coupled. The authors postulate it might be through an integral membrane protein. Discovering this coupling mechanism would be an incredible scientific advance and merit publication with broad readership, but the authors have not done this.

Comments for the author

There are two significant issues with the two proposed conceptual advances in this study. The first lacks novelty and the second is incorrectly interpreted.
1) Cytosolic redox conditions affect ER localized glycosylation.
I would like to turn the author's attention to the background provided in their introduction that illustrates the lack of novelty in their first claim. The author's state:
"Hypoglycosylation of sequons due to disulfide formation can be dependent upon STT3A or STT3B and is reversed when proteins are prevented from forming disulfides under highly reducing conditions (Allen et al., 1995; Cherepanova et al., 2014).
In addition, STT3B-dependent glycosylation of cysteine-proximal sites requires the oxidoreductase activity of the thioredoxin-domain containing subunits MagT1 or TUSC3 (Cherepanova and Gilmore, 2016; Cherepanova et al., 2014)"
These prior studies have already provided evidence that ER redox conditions and disulfide formation directly impact glycosylation efficiency.

Furthermore, the authors continue:
"Exactly how these PDI enzymes are reduced is unknown but recent evidence suggests a role for the cytosolic reductive pathway in correct disulfide formation, driven by the reduction of thioredoxin reductase (Cao et al., 2020; Poet et al., 2017)."
While written in a way that places the focus on PDI enzymes, the main conclusion from Cao et al. and Poet et al. is that cytosolic redox state affects ER redox state—and subsequently disulfide formation.

Considering these prior studies together—rather than separately and in different contexts—classifies this study as a proof of concept. The minute details of hemopexin hypoglycosylation are, in my opinion, not relevant for publication unless the authors can place it into a broader biological context and demonstrate physiological importance.

2) Cytosolic redox state, ER redox state, disulfide quality control, and glycosylation efficiency are coupled through MagT1/TUSC3.
The final piece of evidence implicating MagT1/TUSC3 as a potential coupling mechanism is interpreted incorrectly (Fig. 6B, 6D). To summarize the data, there is an inherent hypoglycosylation of N187 that is rescued through addition of G6-P.
This hypoglycosylation is exacerbated upon STT3B KO and MagT1/TUSC3 KO. However the exacerbated hypoglycosylation can be partially rescued upon addition of G6-P.

© 2021. Published by The Company of Biologists under the terms of the Creative Commons Attribution License (https://creativecommons.org/licenses/by/4.0/).

The author's interpretation is that the cytosolic reductive pathway (as manipulated by G6-P) is important for maintaining optimal STT3B activity. This is entirely incorrect.
Since G6-P addition improves glycosylation in the absence of STT3B/MagT1/TUSC3 this is clear evidence that G6-P is improving glycosylation efficiency through a distinct mechanism. In other words, how can G6-P (cytosolic reductive pathway) be coupled to STT3B if its effects are still observed when STT3B is missing?

In addition, the main link to off pathway disulfide formation is provided in Figure 3B. This experiment is a blunt force approach which can hardly be interpreted. Removing all cysteines from Hemopexin is likely to affect its folding globally, which will inherently affect glycosylation as the two are tightly coupled. This is clear from the authors result in which suddenly there is a major proportion of fully unglycosylated product in their translation extract (~50%).
Using this as evidence to claim off-pathway disulfide formation as the culprit of hypoglycosylation is far-fetched. As this result is the turning point in the study that leads the authors towards reductases as a coupling mechanism, actually demonstrating a link to off-pathway disulfide formation is critical.

Technical issues:
1) All graphs demonstrating "fraction hypoglycosylated" should be normalized with the "H2O" sample equal to 1. This is important because the relevant piece of information is the delta between conditions. For example, in figure 1E it is important to know that there is a 33% reduction in hypoglycosylation upon addition of G6-P.

2) When comparing a minor population in an in-vitro translation extract by radiography (such as the hypoglycosylated band), comparing between gels and relying on historical quantifications is not best practice. Although the radiography is highly sensitive and can be quantitative to a degree, the nature of the assay is very qualitative. Thus, all experiments should contain appropriate controls. For example, Figure 6B should have a wt control with all three conditions run in the same experiment. Similarly, Figure 5B lacks a WT control.

3) Again, related to the fact that the authors are examining a minor band the gel for figure 1D needs to be re-run such that the amounts of fully glycosylated product are qualitatively equal. The dramatic reduction of total product in the G6-P condition makes interpretation of the result extremely difficult and only invites skepticism.

4) In the abstract the authors state "We also show that efficient glycosylation at this site is dependent on STT3A-contatining oligosaccharide transferase". This seems like a typo as figure 6A clearly demonstrates that N187 glycosylation efficiency is not at all altered by STT3A KO.

First revision

Author response to reviewers' comments

Point by point response to reviewer's comments.

All changes to the manuscript text have been highlighted in yellow in the submitted document.

Reviewer 1

We note the positive comments made by the reviewer on the manuscript and comment below on the minor points raised.

1. *The title refers to "complete N-glycosylation" of an acceptor site, the quantification data included in the manuscript show this is rarely if ever achieved in their in vitro system. I suggest the title is changed to "required for efficient N-glycosylation" or some similar term.*

We agree with this point and have changed the title accordingly (line 1).

© 2021. Published by The Company of Biologists under the terms of the Creative Commons Attribution License (https://creativecommons.org/licenses/by/4.0/).

2. *Following on from point 1, can the authors clarify whether existing literature indicates that a fraction of hemopexin is hypoglycosylated in vivo under "normal" conditions, i.e. is it a tricky substrate anyway?*

Hemopexin is indeed hypoglycosylated when expressed in mammalian cells a condition that is exacerbated when STT3B is knocked down or deleted. We have added a phrase on line 106-107 to emphasise this point.

3. *Line 120. The authors should point out that it is a minor fraction of hemopexin that is hypoglycosylated under the conditions described. This fraction varies, but in each case the majority of the translocated protein is fully N-glycosylated.*

We have added a phrase on line 122 to clarify this point.

4. *At various points where the authors state that reagents like G6-P reverse the hypoglycosylation of N-187, this is true in the sense that the level of under-glycosylated material is reduced but it is never completely absent. The term partially reversed would seem to be more appropriate here and indeed the authors use the phrase "partially resolve the hypoglycosylation" in line 286 of their manuscript in a similar context.*

We have removed any reference to complete reversal of hypoglycosylation (see lines 97, 123, 149, 169, 212, 223, 298, 302).

5. *The authors use protease protection to show the circa 50 kDa products is non-translocated precursor protein. Do the authors have any suggestions as to why G6P would reduce the amount of this species. Likewise, in the case of the single cysteine mutant, C188-RC, there is a substantial increase in the amount of this non-translocated species? Is the mutant partially defective in membrane translocation?*

We had noticed this ourselves but were reluctant to draw any firm conclusions. All our samples are prepared by first isolating the SP-cells from the translation and then carrying out an immunoisolation. It may well be that the untranslocated hemopexin is "stickier" when synthesised in the absence of G6-P so is co-isolated with the SP-cells. There were instances where there is little difference between the amount of untranslocated protein in the absence and presence of G6-P (see Fig. 1B, 4, 6A) complicating any interpretation.

6. *Figure 4B. There seems to be a general loss of signal when samples are treated with PK in the presence of G6-P? Any thoughts?*

This is simply due to a lower level of translation in the original translations prior to proteinase K treatment. The important point is that the product seen in Fig. 4A, lane 4 (arrowhead) is lost after proteinase K treatment, identifying this as untranslocated protein.

7. *The data shown in Figure 6 are clear cut and direct us to STT3B complex and its MagT1/TUSC3 subunits. In panel 6B I can see an additional faint band appearing (as compared to panel 6A) which runs between the hypoglycosylated hemopexin and its non-translocated precursor as defined above. Does this mean that an additional sequon is now being partially under-glycosylated when the STT3B complex is defective?*

There is indeed a faint additional band in panel 6B that could be due to further hypoglycosylation in this cell-line. Due to the very faint appearance and inconsistency between experiments we did not want to overinterpret this observation. This additional band is also seen in transfected cells (See Fig 1C, Shrimal and Gilmore 2013)

8. *I would encourage the authors to include a simple schematic or cartoon that outlines the two proposed potential fates of N-187 when a disulphide is formed before or after its arrival at the STT3A complex. I think this would be very helpful for many readers.*

© 2021. Published by The Company of Biologists under the terms of the Creative Commons Attribution License (https://creativecommons.org/licenses/by/4.0/).

We have included an additional cartoon (figure 7) for clarification, added a figure legend and altered the discussion (lines 261-265) to refer to the figure.
Reviewer 2

There are two significant issues with the two proposed conceptual advances in this study. The first lacks novelty and the second is incorrectly interpreted.

1) *Cytosolic redox conditions affect ER localized glycosylation. I would like to turn the author's attention to the background provided in their introduction that illustrates the lack of novelty in their first claim. The author's state: "Hypoglycosylation of sequons due to disulfide formation can be dependent upon STT3A or STT3B and is reversed when proteins are prevented from forming disulfides under highly reducing conditions (Allen et al., 1995; Cherepanova et al., 2014). In addition, STT3B-dependent glycosylation of cysteine-proximal sites requires the oxidoreductase activity of the thioredoxin-domain containing subunits MagT1 or TUSC3 (Cherepanova and Gilmore, 2016; Cherepanova et al., 2014)". These prior studies have already provided evidence that ER redox conditions and disulfide formation directly impact glycosylation efficiency. Furthermore, the authors continue: "Exactly how these PDI enzymes are reduced is unknown but recent evidence suggests a role for the cytosolic reductive pathway in correct disulfide formation, driven by the reduction of thioredoxin reductase (Cao et al., 2020; Poet et al., 2017)." While written in a way that places the focus on PDI enzymes, the main conclusion from Cao et al. and Poet et al. is that cytosolic redox state affects ER redox state—and subsequently disulfide formation. Considering these prior studies together—rather than separately and in different contexts—classifies this study as a proof of concept. The minute details of hemopexin hypoglycosylation are, in my opinion, not relevant for publication unless the authors can place it into a broader biological context and demonstrate physiological importance.*

The reviewer makes a valid point regarding the potential novelty of the work. However, we consider demonstrating a link between the cytosolic reductive pathway and glycosylation site occupancy to be an important advance. The previous work published by us, and others covers the general principle of a link between disulfide formation and glycosylation and then, separately, a link between the cytosolic reductive pathway and resolution of non-native disulfides in the ER. Showing that the oxidoreductases that are part of the glycosylation machinery require a robust cytosolic reductive pathway is significant as this level of detail has not been determined previously. Our understanding of the reductive pathway required for correct disulfide formation is still poor and while the cytosolic pathway may well be required, we do not yet know if there are other pathways that work in parallel. Just like the Ero1 pathway for oxidation of disulfides is not the only pathway for de novo disulfide formation, there could be other systems that act to reverse thiol oxidation. Hence, the observations in this paper add an additional layer of detail not presently in the literature.

2) *Cytosolic redox state, ER redox state, disulfide quality control, and glycosylation efficiency are coupled through MagT1/TUSC3. The final piece of evidence implicating MagT1/TUSC3 as a potential coupling mechanism is interpreted incorrectly (Fig. 6B, 6D). To summarize the data, there is an inherent hypoglycosylation of N187 that is rescued through addition of G6-P. This hypoglycosylation is exacerbated upon STT3B KO and MagT1/TUSC3 KO. However, the exacerbated hypoglycosylation can be partially rescued upon addition of G6-P. The author's interpretation is that the cytosolic reductive pathway (as manipulated by G6-P) is important for maintaining optimal STT3B activity. This is entirely incorrect. Since G6-P addition improves glycosylation in the absence of STT3B/MagT1/TUSC3, this is clear evidence that G6-P is improving glycosylation efficiency through a distinct mechanism. In other words, how can G6-P (cytosolic reductive pathway) be coupled to STT3B if its effects are still observed when STT3B is missing?*

We are grateful to the reviewer in highlighting our inability to express our conclusions clearly. We did not mean to suggest that only STT3B activity is affected by the cytosolic reductive pathway. Following advice from both reviewers we have made this clearer both in the results (lines 255-257), the discussion (lines 261-265) and with the inclusion of figure 7.

© 2021. Published by The Company of Biologists under the terms of the Creative Commons Attribution License (https://creativecommons.org/licenses/by/4.0/).

3) *In addition, the main link to off pathway disulfide formation is provided in Figure 3B. This experiment is a blunt force approach which can hardly be interpreted. Removing all cysteines from Hemopexin is likely to affect its folding globally, which will inherently affect glycosylation as the two are tightly coupled. This is clear from the authors result in which suddenly there is a major proportion of fully unglycosylated product in their translation extract (~50%). Using this as evidence to claim off-pathway disulfide formation as the culprit of hypoglycosylation is far-fetched. As this result is the turning point in the study that leads the authors towards reductases as a coupling mechanism, actually demonstrating a link to off-pathway disulfide formation is critical.*

The main link to off-pathway disulfide formation is in Fig. 4A and Figure 3B was simply our attempt to determine why N187 was still hypoglycosylated in the absence of the cognate partner for C188 i.e., C200. Indeed, removing all thiols was a blunt approach but it did show that if only C188 is present then N187 is not hypoglycosylated. Hence, it is likely that C188 forms a non-native disulfide when C200 is mutated. The presence of untranslocated material in the samples has been explained above in response to reviewer 1 (point 5). This construct seems particularly “sticky” and gives a higher level of background (untranslocated) material. This does not detract from the specific point regarding the hypoglycosylation of N187 in the C200 mutant.

Technical issues:

1) *All graphs demonstrating “fraction hypoglycosylated” should be normalized, with the “H2O” sample equal to 1. This is important because the relevant piece of information is the delta between conditions. For example, in figure 1E it is important to know that there is a 33% reduction in hypoglycosylation upon addition of G6-P.*

We had considered several ways to present the data and eventually decided to keep with previous publications and calculate a “fraction hypoglycosylated”. This allows the reader to evaluate the overall level of hypoglycosylation which would be lost if we normalised the data to the “H_2O” sample. This would be particularly important for figure 6 as this overall level of hypoglycosylation is different between the cell-lines analysed so we would not want to lose this valuable information. We would also lose any error bars for the “H_2O” sample if we normalised. For this reason, we would prefer to stay with the quantification as originally presented.

2) *When comparing a minor population in an in-vitro translation extract by radiography (such as the hypoglycosylated band), comparing between gels and relying on historical quantifications is not best practice. Although the radiography is highly sensitive and can be quantitative to a degree, the nature of the assay is very qualitative. Thus, all experiments should contain appropriate controls. For example, Figure 6B should have a wt control with all three conditions run in the same experiment. Similarly, Figure 5B lacks a WT control.*

All our quantifications were carried out using phosphorimage analysis which is linear over several orders of magnitude. We found that the level of hypoglycosylation wild-type cells (either HEK293 or HT1080) was very consistent at between 20-25% over multiple experiments and over several months during which this work was carried out. In addition, we always included the wild-type controls when carrying out experiments with KO cells which were carried out on the same day and run on the same gels. We simply omitted these lanes during figure preparation as they seemed superfluous and led to a cluttering of already complex figures. We include these below and are happy to include as supplementary data but would ask the reviewer to consider whether they add anything to the validity of the data as presented originally.

© 2021. Published by The Company of Biologists under the terms of the Creative Commons Attribution License (https://creativecommons.org/licenses/by/4.0/).

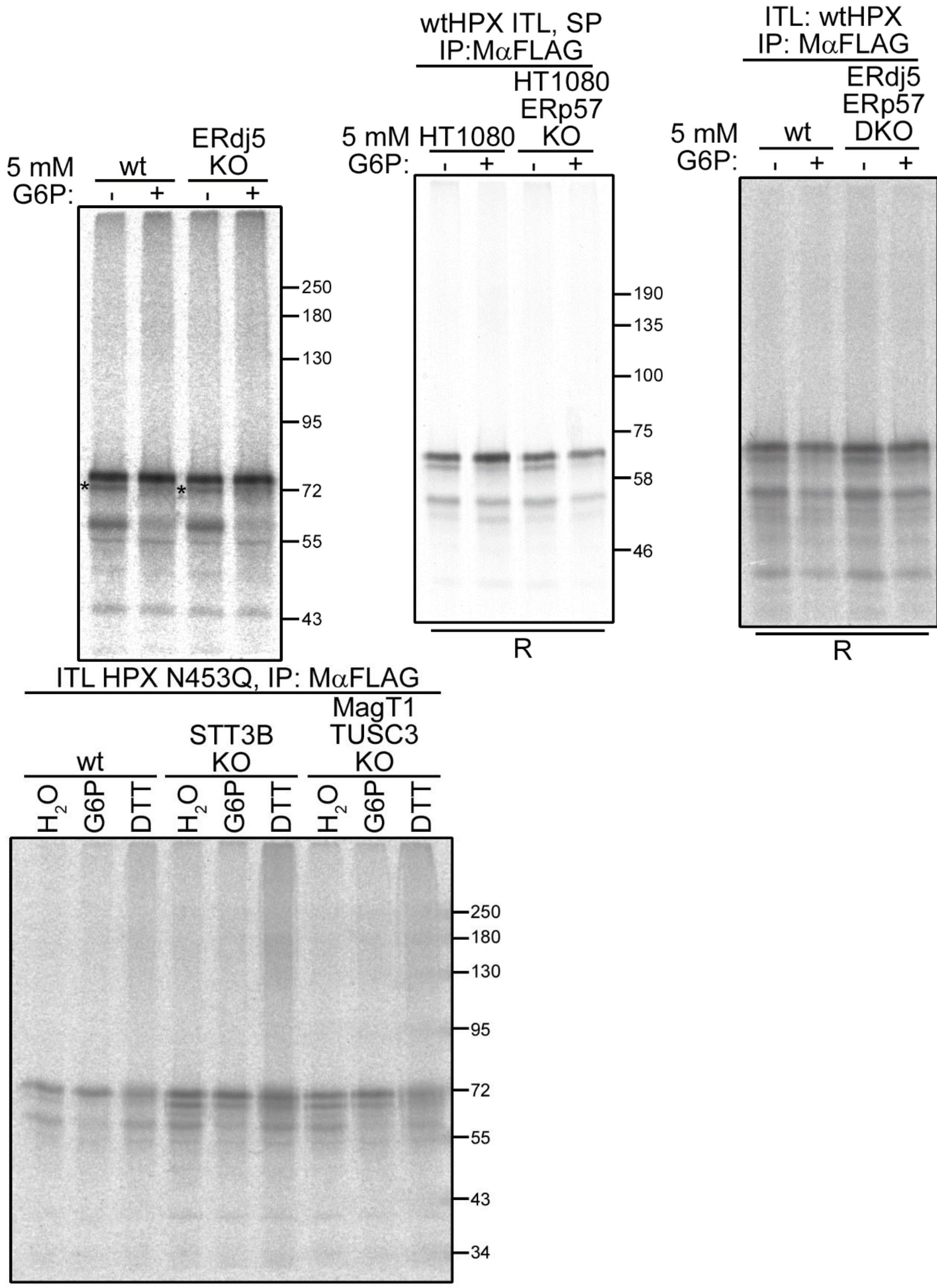

3) *Again, related to the fact that the authors are examining a minor band, the gel for figure 1D needs to be re-run such that the amounts of fully glycosylated product are qualitatively equal. The dramatic reduction of total product in the G6-P condition makes interpretation of the result extremely difficult and only invites scepticism.*

We have replaced figure 5D with another experiment that has more equal loadings of the samples. The experiment was carried out three times for the quantification seen in 1E. Note the statistical significance of the differences in fraction hypoglycosylation seen following inclusion of G6-P or NADPH.

© 2021. Published by The Company of Biologists under the terms of the Creative Commons Attribution License (https://creativecommons.org/licenses/by/4.0/).

4) *In the abstract the authors state "We also show that efficient glycosylation at this site is dependent on STT3A-contatining oligosaccharide transferase". This seems like a typo as figure 6A clearly demonstrates that N187 glycosylation efficiency is not at all altered by STT3A KO.*

This statement was correct, and we have now changed it to make the point clearer (lines 29-31).

Second decision letter

MS ID#: JOCES/2021/259340

MS TITLE: A Cytosolic Reductase Pathway is Required for Efficient N-Glycosylation of an STT3B-Dependent Acceptor Site.

AUTHORS: Marcel van Lith, Marie Anne Pringle, Bethany Fleming, Giorgia Gaeta, Jisu Im, James Reid Gilmore, and Neil J Bulleid
ARTICLE TYPE: Research Article

I am happy to tell you that your manuscript has been accepted for publication in Journal of Cell Science, pending standard ethics checks.

© 2021. Published by The Company of Biologists under the terms of the Creative Commons Attribution License (https://creativecommons.org/licenses/by/4.0/).
